# Understanding inequities in accessing neurodevelopmental pathways: a multiple methods study investigating healthcare professionals’ views on access for minoritised ethnic families

**DOI:** 10.1136/bmjment-2026-302716

**Published:** 2026-08-11

**Authors:** Sarah Elaine Cooper, Shireen Patel, Camilla Babbage, Kapil Sayal, Madeleine Jane Groom, Jennifer Logan, Vibhore Prasad, Charlotte L Hall

**Affiliations:** 1 Nottinghamshire Healthcare NHS Foundation Trust, Institute of Mental Health, Nottingham, UK; 2 Applied Research Collaboration East Midlands (ARC EM), University of Nottingham, National Institute for Health and Care Research, Nottingham, UK; 3 MindTeach HealthTech Research Centre, School of Medicine, University of Nottingham, National Institute for Health and Care Research, Nottingham, UK; 4 School of Medicine, Institute of Mental Health, Academic Unit of Mental Health and Clinical Neurosciences, University of Nottingham, Nottingham, UK; 5 Nottingham Biomedical Research Centre, University of Nottingham, National Institute for Health and Care Research, Nottingham, UK; 6 Strategy, Transformation and Commercial Development, South West London and Saint George’s Mental Health NHS Trust, London, UK; 7 School of Medicine, University of Nottingham, Nottingham, UK

**Keywords:** Neurodevelopmental Disorders, Mental Health, Mental Health Services

## Abstract

**Background:**

Minoritised ethnic families in England experience disproportionate barriers to accessing neurodevelopmental disorder (NDD) assessment and support despite universal healthcare provision. Existing evidence has focused on caregivers’ perspectives or non-UK settings but little is known about how healthcare professionals (HCPs) perceive and interpret these inequities. Addressing this gap is essential for improving access, service design and workforce development.

**Objective:**

To explore HCPs’ perceptions of the barriers and facilitators influencing minoritised ethnic families’ access to NDD services in England.

**Methods:**

A multiple methods design was used, comprising an online national survey (n=264) and two online focus groups with primary, secondary and allied health professionals (n=9). Survey data were summarised descriptively and free text responses analysed using summative content analysis. Focus group transcripts underwent reflexive thematic analysis. Findings were summarised following parallel analysis of each data set. Patient and public involvement and engagement members from minoritised ethnic backgrounds contributed throughout.

**Findings:**

HCPs identified four interconnected barriers they perceived to influence access for minoritised ethnic families: (1) language, communication and meaning-making challenges, including differences in how concepts and expressions are understood, inconsistent interpretation and limited mental health literacy; (2) difficulties navigating complex, fragmented NDD pathways, exacerbated by digital literacy demands and unclear referral routes; (3) limited caregiver knowledge and understanding of NDDs, affecting symptom recognition and the ability to advocate effectively and (4) cultural norms, beliefs and stigma, including preferences for non-Western models of care, fear of diagnostic labels and the influence of extended family. Professionals also highlighted the impact of unconscious bias and limited cultural representation within services on referral decision-making and caregiver engagement. HCPs described several effective strategies for mitigating these barriers, such as culturally attuned communication, community-based engagement and flexible assessment approaches.

**Conclusions:**

Inequitable access to NDD services arises from the interaction of cultural, relational and structural factors across the healthcare pathway. HCPs’ insights reveal multiple entry points for improving equity, particularly through communication support, culturally responsive practice and simplified referral processes.

**Clinical implications:**

Embedding culturally informed communication, improving caregiver education, strengthening workforce diversity and coproducing service changes with communities may reduce avoidable delays and promote equitable access to neurodevelopmental assessment and support.

**WHAT IS ALREADY KNOWN ON THIS TOPIC**

- Minoritised ethnic families face additional barriers to accessing neurodevelopmental services. However, little is known about how healthcare professionals (HCPs) themselves understand these barriers within neurodevelopmental disorder (NDD) pathways.

**WHAT THIS STUDY ADDS**

- This study provides the first national evidence on HCPs’ views of barriers affecting minoritised ethnic families’ navigation of NDD pathways in England.

**HOW THIS STUDY MIGHT AFFECT RESEARCH, PRACTICE OR POLICY**

- The findings highlight actionable system-level changes and the importance of community partnership. Further research should directly capture families’ experiences and evaluate interventions to reduce inequities.

## Background

 Neurodevelopmental disorders (NDDs), including autism spectrum disorder, attention-deficit/hyperactivity disorder (ADHD) and tic disorders, are common, typically emerge in childhood and are frequently lifelong.^1 2^ NDDs can affect educational attainment, social functioning and family well-being, and early identification and intervention are associated with improved long-term outcomes.^2–5^ However, prevalence, recognition and access to diagnosis vary across sociodemographic groups.^3 6 7^ In England, access to NDD assessment is typically initiated via a general practitioner (GP) or other healthcare professionals (HCPs), with onward referral to child and adolescent mental health services (CAMHS), paediatrics or specialist neurodevelopmental pathways. Service configuration varies widely across regions, resulting in inconsistent pathways and provision.^8^ Families commonly encounter barriers including long waiting times, unclear referral criteria, limited-service capacity and fragmented care.^9 10^

The population in England and Wales is ethnically and culturally diverse, with 25.6% identifying as belonging to an ethnic group other than white British. The most commonly self-identified non-white ethnicities were Indian, Pakistani and Black African.^11^ However, these groups collectively form part of the Global Majority,^12^ reflecting populations that are numerically dominant worldwide but structurally minoritised in Western settings.

While many barriers to healthcare are shared across populations, evidence suggests that minoritised ethnic individuals experience additional and distinct challenges when accessing healthcare,^13^ including stigma, language and cultural barriers and mistrust arising from experiences of discrimination. These contribute to inequities in access despite universal healthcare provision and equality legislation in England.^13 14^ Consequently, minoritised ethnic people continue to face difficulties accessing NDD services.^15^

To date, much of the evidence exploring experiences of accessing care for minoritised ethnic people originates from studies conducted outside England. International research remains informative, particularly within western high-income contexts that share comparable dynamics of racialisation and service inequity. Although healthcare systems in these western contexts differ, many share common features, such as reliance on professional referral pathways and culturally framed diagnostic practices.^16^ As such, examining HCPs’ perspectives within the English context may offer insights relevant to other comparable western healthcare systems.^17^ In contrast, findings from non-Western or Global Majority countries may be less transferable due to differing sociopolitical drivers shaping access.^16^

In the UK, research demonstrates that caregivers (eg, parents) being from a minoritised ethnicity negatively predicts access to early support (across education, health and social care) for children and young people (CYP) with diagnosed or suspected neurodivergence.^18^ Consistent with this, CYP from minoritised ethnic groups are less likely to access CAMHS than their white British peers.^19^ A scoping review identified barriers to accessing neurodevelopmental services for ethnic minority carers in the UK, including language, stigma, mistrust in healthcare systems and structural factors such as inconsistent support and negative interactions with professionals.^20^ Facilitators included culturally tailored communication and community-based support approaches.^20^ Although one study explored professionals’ experiences of supporting South Asian families with disabilities,^21^ it was linked to one broad ethnic group and a broader category of disability rather than NDD specifically. To support interpretation of the findings, this study is informed by a socioecological framework of healthcare access, which conceptualises inequities as arising from interacting influences at multiple levels, including individual, interpersonal, community and structural factors.^22^

To our knowledge, no study has directly examined how HCPs conceptualise and navigate barriers faced by minoritised ethnic people across NDD pathways in England. This represents a critical gap, given clinicians’ central role in referral decisions, assessment practices and shaping families’ access to support.

## Objective

This study addresses the evidence gap by examining HCPs’ perceptions of barriers to accessing NDD services for minoritised ethnic CYP and families in England. Understanding these perspectives is essential for informing equitable service design, workforce development and policy initiatives within England and comparable Western healthcare systems.

## Methods

A multiple-methods approach to analysis allowed for the integration of qualitative data from both open text survey responses and focus group data.^23^ The datasets comprised an online survey and two focus groups, one for primary HCPs and one for secondary and allied health professionals. Findings are reported using the Good Reporting of a Mixed Methods Study checklist^24^ as a guide (see online supplemental file 1). Initially, the numerical survey data are presented ahead of open text survey data, then focus group themes are shared. The section finishes with a summary integrating the findings. Ethical approval was granted on 14 May 2024 by West Midlands – Edgbaston Research Ethics Committee and the Health Research Authority (reference number 24/WM/0063). This work formed part of the Exploring the Experiences of Accessing services and understanding Neurodevelopmental Disorders for ethnic minorities in England (EXPAND) study, funded by the National Institute for Health and Care Research (NIHR) under its research for patient benefit programme (grant Reference Number NIHR206134). EXPAND aimed to explore the barriers faced by minority ethnic families in England accessing NDD healthcare using the experiences and perspectives of caregivers, CYP, HCP and educational professionals. Findings were interpreted using a socioecological framework to examine how barriers operate across individual, interpersonal, community and structural levels.^22^

### Online survey

#### Design and instrument

This national survey was hosted on research electronic data capture (REDCap) (https://project-redcap.org/).^25^ The survey comprised up to 43 items navigated via in-built survey logic, allowing relevant follow-up questions based on previous answers to appear, including items on the following topics: screening (four), demographic (eight), professional background (nine) and experiences of working with minoritised ethnic families (21). All sections were mandatory. The survey was co-developed with lived experience experts (patientnt and Public Involvement and Engag and public involvement and engagement (PPIE)), primary and secondary HCPs and research experts in the field. The survey was piloted by the study team and PPIE members prior to the start and reported in line with the Checklist for Reporting Results of Internet E-Surveys^26^ (see online supplemental file 1). REDCap automatically assigned participants’ unique identifiers, and email verification prevented duplicated (n=5) and non-genuine responses and was open between 9 August 2024 and 20 May 2025.

#### Procedure

Participants were recruited via study adverts disseminated via: (1) The UK NIHR Research Delivery Network; (2) Contact, Help, Advice and Information Network, an online community for HCPs; (3) professional connections of the study team.

Prior to accessing survey questions, potential participants were directed to the participant information sheet and screening questions. Eligible participants were directed to the consent form. Participation was voluntary with an optional entry into a prize draw to receive a gift voucher.

#### Participants

Eligible participants had experience in providing mental healthcare (eg, GPs, CAMHS/paediatric clinicians, clinical psychologists, occupational therapists and speech and language therapists) and with CYP from minoritised ethnic backgrounds. 713 professionals accessed the survey link; of these, 352 (49.4%) engaged in the screening process. 330 (93.8%) potential participants passed the screening process and, of those who were eligible to take part, 264 (80.0%) went on to fully complete the survey. Only data from these 264 completed surveys were included in the analysis.

### Qualitative focus group

#### Instruments

The focus group topic guide (online supplemental file 2) was informed by the survey findings, consultations with professionals, PPIE and relevant literature.^27 28^ Prompts were included to facilitate discussion.

#### Participants

77 survey participants expressed interest in focus group participation at the end of the online survey, of whom 11 (14.3%) consented and nine attended (11.7%). Participants comprised both female (n=6) and male (n=3) HCPs from primary care and paediatric services who had previously completed the online survey.

#### Procedure

Interested participants received an email with session details and an online consent form. Two online semi-structured focus groups (n=5 lasting 57 min and n=4 lasting 66 min) were conducted via Microsoft Teams, recorded and transcribed. Discussions covered challenges in assessing and diagnosing minoritised ethnic families, service adaptations, representation and barriers to access (see online supplemental file 2).

#### Analysis

Quantitative survey data were exported to Microsoft Excel and summarised using descriptive statistics (percentage/frequency, mean and range). Descriptive comparisons between different groups explored similarities or differences in perceptions. Open-ended survey responses were analysed using summative content analysis.^29^ SEC identified key terms and concepts, discussed these with CB and the PPIE group and developed an initial coding scheme. This was refined with CB, SP and CLH and applied to all free text responses.

Focus group transcripts were automatically generated by Microsoft Teams and anonymised and checked for accuracy by SEC. The focus groups were analysed using reflexive thematic analysis following Braun and Clarke’s six phases^30^. A realist-essentialist approach was used to prioritise participants’ explicit accounts (semantic-level coding), rather than developing latent interpretations, consistent with the study aim of capturing HCPs’ perspectives as expressed.^31^ SEC led coding and initial theme development, with regular discussions with CB, SP and CLH to refine codes and themes across the analytic phases. PPIE contributors informed interpretation through review and refinement of themes. Reflexivity was maintained through ongoing team discussions, drawing on researchers’ positionality to critically reflect on how assumptions may shape analysis.

A parallel analysis approach was applied to the qualitative and quantitative findings,^32^ with each dataset being analysed separately and then combined to provide a comprehensive overview.^23^

#### Reflexive statement

Researcher positionality informed data collection and analysis.^33^ SEC (British cisgendered female of mixed Black Caribbean/white ethnicity) led the focus groups, transcription and coding and has a background in secondary education and lived experience of parenting a child with special educational needs. SEC’s positionality as both an insider and outsider informed their approach to data analysis and interpretation. Collaboration with the wider team was crucial to avoid assumptions being made based on their positionality. CLH (cisgendered, white British female) co-delivered the focus groups and has extensive experience in neurodevelopmental research. CB is a white British cisgendered early career researcher who has been working in research with children, young people and families with mental health and neurodevelopmental conditions for 10 years. SP is a British Indian cisgender early-career researcher with extensive experience in mental health research, particularly focused on addressing health inequalities. The wider team comprises experienced UK-based researchers from diverse ethnic backgrounds and expertise in NDDs, mental health and qualitative research.

### Patient and public involvement and engagement

PPIE was integrated throughout the study design, conduct and analysis. Eight PPIE members from minoritised ethnic backgrounds, with lived experience as parents or caregivers of CYP with NDD, contributed to developing the research questions, survey content and focus group topic guide. PPIE supported reflexive discussions around theme development, ensuring that interpretations reflected community-relevant perspectives and avoided a deficit-focused narrative. PPIE is reported according to The Guidance for Reporting Involvement of Patients and the Public (GRIPP2)^34^ (online supplemental file 1).

## Findings

Initially, the numerical survey data are presented ahead of open text survey data, then focus group themes are shared. The section finishes with a summary synthesising the findings.

Although the data primarily highlight the barriers that HCPs perceived to be experienced by minoritised ethnic families, HCPs also proposed potential solutions to these perceived challenges, presented separately.

### Numerical survey data

The final sample characteristics aligned with The National Health Service (NHS) England workforce profile on gender and ethnic composition^35^ (see online supplemental file 3, table 1).

Participants were employed in a range of mental health services for CYP (see online supplemental file 3). The most common roles were assessment (72.0%) and screening (63.6%), with many HCPs delivering multiple functions. Most respondents were frontline clinicians (92%).

HCPs identified multiple barriers that they perceived were faced by both minority ethnic and white caregivers, though these were reported much more frequently for ethnic minority families. The most frequently cited perceived barriers for minoritised ethnic families were language (90.5%), stigma (86.0%), difficulty navigating services (84.8%), discrimination from family (76.1%) and challenges recognising NDD symptoms (72.7%). Only logistical barriers showed similar prevalence (defined as within 10%) across groups (62.1% vs 56.8%). Full results are shown in table 1.

**Table 1:** Perceived barriers faced by minoritised ethnic caregivers compared with white caregivers when accessing healthcare services for NDD, identified by survey participants

Perceived barriers identified by healthcare professionals* n=264	Number (%) of professionals indicating this barrier for caregivers	
	Minority ethnic caregivers	White caregivers
Language	239 (90.5)	19 (7.2)
Stigma	227 (86.0)	95 (36.0)
Discrimination from family	201 (76.1)	72 (27.3)
Religious beliefs	146 (55.3)	36 (13.6)
Understanding how to access services	224 (84.8)	127 (48.1)
Discrimination from staff/services	113 (42.8)	39 (14.8)
Understanding how to identify symptoms	192 (72.7)	122 (46.2)
Distrust in healthcare services	125 (47.3)	92 (34.8)
Logistics	164 (62.1)	150 (56.8)

* Categories were not mutually exclusive, so multiple barriers were identified by healthcare professional participants.

NDD, neurodevelopmental disorder .

Additional descriptive comparisons between groups showed a similar pattern. Comparing GP versus other HCP perceptions on barriers faced, both groups consistently ranked the same top four barriers that HCP considered the most common (language, stigma, understanding service access and discrimination from family) (see online supplemental file 3). When comparing length of service, HCP with 7+ years’ service versus those with six or fewer years identified the same top four commonly perceived barriers(online supplemental file 3).

### Open text survey data

The survey’s five open-ended questions (online supplemental file 2) provided 454 responses organised into six distinct categories: (1) challenges related to cultural and religious beliefs about NDDs and mental health; (2) the need for clearer, tailored communication; (3) mixed experiences of community engagement; (4) the importance of relationships and continuity of care; (5) limitations and quality concerns in translation/interpretation services; (6) service constraints that restrict personalised, culturally responsive care.

Respondents emphasised the need for greater cultural competence within the HCP workforce, improved support for communication and more meaningful engagement with diverse communities. See online supplemental file 4 for a summary of findings.

### Focus group themes

Reflexive thematic analysis of the focus group data produced four main themes and four subthemes (see table 2).

**Table 2:** Themes and subthemes from focus group thematic analysis

Theme	Description	Subtheme
Perceptions, unconscious bias and greater cultural representation by healthcare professionals is needed to foster trust and encourage engagement from minority ethnic caregivers	Theme 1 illustrates how healthcare and NDD diagnosis when framed through western-focused diagnostic criteria shape perceptions and experiences. This theme also explores how unconscious bias influences how minority ethnic CYP and their families are perceived.	N/A
Culture and community influence perceptions about NDDs and accessing services for minority ethnic caregivers.	Theme 2 presents healthcare professionals’ perceptions on how caregivers, families and the wider community view NDDs and how these views are influenced by culture and religion. Healthcare professionals expressed thoughts on how some NDDs were perceived to be more acceptable than others and how blame is often attributed according to cultural norms.	2a. Cultural constructs of children’s difficulties and help-seeking
Difficulties in communicating about and understanding NDDs created barriers to accessing support.	Theme 3 highlights how language and its use in healthcare settings can create barriers for minoritised ethnic families when seeking support. Differing cultural perspectives on mental health can hinder a minority ethnic family’s ability to communicate mutual meaning. Where there is limited knowledge or understanding of NDDs, this creates further difficulty in communicating needs. The use of translators or interpreters can also contribute to misunderstandings between families and healthcare professionals.	3a. Understanding or knowledge of NDDs in minority ethnic communities hinders communication about a child’s needs 3b. When English is an additional language or translation services are needed.
Lack of awareness of healthcare services and how to navigate them	Theme 4 explores healthcare professionals’ experiences and perceptions of the difficulties minority ethnic families face when seeking out or navigating healthcare services. Healthcare professionals reflected on the difficulties some minority ethnic families had in seeking out and requesting help along with different expectations of the role of healthcare professionals when seeking support.	4a. Cultural norms when interacting with healthcare professionals

CYP, children and young people; NDD, neurodevelopmental disorders.

### Theme 1: perceptions, unconscious bias and greater cultural representation by HCPs is needed to foster trust and encourage engagement from minority ethnic caregivers

HCPs reflected how unconscious biases and assumptions shape the referral pathway for minority ethnic CYP. Participants described the implicit ‘gatekeeping’ or overshadowing role whereby behaviours were sometimes attributed to cultural background or migration experiences, resulting in NDD indicators being overlooked.

People [Healthcare and Educational professionals] will think, oh, well, it’s because they’re you know, they’re maybe a refugee or asylum seeker that’s why they’re not talking as well or not doing things as well, you know, unconscious bias, I feel that sometimes comes into play. (ID61)

Some HCPs felt these biases led to CYP being labelled as ‘naughty’ or managed punitively rather than assessed for NDDs. Limited or unrenewed cultural competency training was seen as a key barrier to addressing these issues. As one HCP reflected on their experience: *“*It’s like once [diversity and inclusion training] when we first joined the Trust and that’s kind of, it” (ID613).

Greater representation within services was viewed as vital for fostering trust and engagement. Staff who shared families’ cultural backgrounds were perceived as better able to explain NDDs, bridge cultural misunderstandings and improve communication.

[Black] Parents won’t stop and talk to me because I’m not part of their community. I’m not trusted. So, I think that absolutely, engaging with the community, the culture, and finding people that can champion within it. (ID167)

### Theme 2: culture and community influences perceptions around NDDs and accessing services for minority ethnic caregivers and families

HCPs reported that cultural and religious beliefs strongly influenced how minority ethnic families interpreted CYP difficulties, which could be impacted by cultural expectations of NDDs (theme 2a), and whether they sought healthcare support.

HCPs noted that in some communities, mental health concerns were seen as something to be managed within the family rather than through healthcare services.

I think it’s very frowned upon and more kind of within the families and (community) and the ideas within the families tend to be ‘no, you need to go out, be hard working and… crack on’. It’s not really seen as something that you should be seeking help and advice for. (ID18)

This was different to physical health, where going to seek advice from your doctor was more accepted.

But culturally, you don’t go to the GP until you have a temperature, you’re coughing, you can’t talk or your leg is broken. So, who is gonna go to hospital because their child is not sleeping? You know, it’s not part of what the people do culturally. (ID638)

Some HCPs reported that in certain minoritised ethnic communities, western medicine was not always seen as the most appropriate approach. Caregivers were described as preferring traditional cultural or religious treatments, particularly among South Asian families: “Indian families, particularly for mental health issues, they will probably prefer going to astrologers or priests, sort of seeking their advice before going to any doctors or anybody, they prefer that more” (ID74)*.*

Stigma, especially from extended family members, reinforced shame and delayed help seeking:

I think for some people they sense it as somehow being their fault and also brings shame to the family. (ID167)

#### Theme 2a: cultural constructs of children’s difficulties and help-seeking

HCPs reflected that cultural expectations of CYP behaviour sometimes discouraged parents from recognising neurodevelopmental needs.

I think cultural expectation it is quite a bit of a barrier. I always have to think about the cultural expectation of children’s behaviour. (ID522)

These cultural expectations included ideas around how CYP should behave in public places, at family or community events and the importance of CYP being quiet, polite or deferential to their elders or being ‘seen but not heard’. These expectations often influenced how caregivers understood or perceived a child’s behaviour. HCPs felt that at times some caregivers saw the NDD as an excuse for poor behaviour and not a healthcare need.

It was quite difficult for parents to kind of understand… that she’s not doing it to be rude. There was, … a respect your elders… attitude at the house. She’s doing it because of this… neurodiversity that she’s got. (ID613)

Some HCPs described caregivers rejecting diagnoses and disengaging from services due to fears that diagnostic labels could expose their child to stigma, discrimination or limit future opportunities.

I think the rejection of the label, the rejection of the service and the support sometimes comes from them thinking, if I accept this label, then my child will never be able to achieve anything in life. (ID638)

HCPs also noted that they often found that some diagnoses were perceived as more acceptable.

The family [were] much more accepting of an ADHD diagnosis and it did kind of link back to the what like their family would think and what people might say, and it’s kind of seen that ADHD was a little bit more kind of socially acceptable compared to autism for this family. (ID613)

### Theme 3: difficulties in communicating about and understanding NDDs created barriers to accessing support

Language differences, health literacy and communication gaps created barriers to accessing support. Communicating meaning around a child’s needs in healthcare settings was often seen as a challenge for many minoritised ethnic caregivers. Difficulties in communication arose when English was an additional language but also when understanding of NDDs was limited and caregivers struggled to identify or explain their child’s symptoms or behaviour (theme 3a). As one GP explained: *“*In my experience they can’t express the kind of thing they need to express so that delays the whole process” (ID74).

Differences in how language is used across cultures also created potential for miscommunication. One HCP explained that:

the use of English is different from culture to culture. So, you say things like a child is ‘full of beans’, where I come from that doesn’t mean anything. (ID638)

Additionally, when interpreters or translators were used to address language barriers, HCPs reported that misunderstandings were commonly encountered (theme 3b).

#### Theme 3a: understanding or knowledge of NDDs in minority ethnic communities hinders communication about a child’s needs

HCPs reflected that some caregivers did not actively seek NDD support for their child as they lacked knowledge about NDDs, leading to misattribution of a child’s difficulties (eg. stubbornness or defiance) and therefore not considering NDD as a possible explanation:

They don’t understand what ADHD is in the first place, so they don’t think that child is unwell, so when that child is struggling, they just think that child is stubborn, or he doesn’t listen. (ID638)

HCPs also noted that when caregivers are unfamiliar with the level of information required, struggled with language to communicate need or were unable to recognise or explain their child’s symptoms, it made it difficult for them to gather the necessary information and deliver effective care.

They [ethnic minority parents] just bring the child [and do not explain anything]. That makes it a bit tricky for us to provide the level of support in a way, because when we're doing assessment, we would like to know the necessary information in order to provide that support and by them not knowing or not being familiar with what is required. (ID522)

#### Theme 3b: when English is an additional language, the use of translators or interpreters can create misunderstandings between families and HCPs

HCPs consistently reported challenges when working with families for whom English was not a first language, describing improvising questions with meaning often lost in translation:

When initially investigating with some of these populations, there are no sort of standardised kind of questions for that so you have to improvise. Yeah, the things can get lost in translations. (ID74)

There was noted difficulty in access to translation services but when they were available, HCPs also reported a lack of confidence in interpretation accuracy.

Relying on an interpreting service is really difficult you don’t know what’s being said and you feel sometimes what you’ve said has not been interpreted appropriately. (ID61)

### Theme 4: lack of awareness of healthcare services and how to navigate them

HCPs described how limited system knowledge, digital literacy requirements and complex pathways hindered families’ access.

HCPs reflected that the shift to caregiver-led referrals and online forms created additional barriers for some minoritised ethnic families:

So some of the systems now requires parents to do the referral themselves, which is great for GPs because it saves us time but you know that does require you to be literate and digitally literate and have the time and wherewithal to fill out forms and speak the language and all the rest of it, which is quite a barrier to some people. (ID61)

Even when self-referral was not required, navigating NDD services was described as highly complex even for those working in them. As one HCP noted: “navigating CAMHS as a whole and the neurodiversity service is just like a complete minefield that even myself as a practitioner who works on CAMHS, I can’t fully understand it” (ID613).

HCPs felt families’ limited understanding also led to misconceptions about service eligibility and cost, resulting in unmet need and delayed care.

I’ve met people who did not escalate their children’s behaviour because they thought they needed to pay. (ID638)

#### Theme 4a: cultural norms when interacting with HCPs

HCPs reflected that deferential cultural norms left some families less able to advocate for their child’s needs in a system that rewards assertiveness and persistence.

in some countries there is still a more paternalistic way of seeing a doctor and when we ask patients about their ‘expectations’ and what they would like to happen they sometimes find this a bit strange, as ‘we are the doctor and should know what to do. (ID152)

Reluctance to challenge professionals or ask for clarification can hinder accurate assessment and delay access to services, with HCPs noting that patients often felt *‘too shy*’ (ID638) to say they did not understand. Within an overstretched system, assertive advocacy was described as a key determinant of access (rather than the child’s need), creating inequities whereby confident, articulate and system-literate caregivers were more likely to secure support.

You have to be assertive, because this is such an overloaded service and there’s massive waiting lists and it’s very easy for people to have an initial assessment and then sort of get signposted somewhere else. The few that managed to get through the many barriers that are in their way do have to be articulate, educated with an agenda and assertive. (ID61)

### Summary of findings

Post-analysis combination of the free text and focus group data identified four overarching categories of barriers shaping minoritised ethnic families’ access to NDD services: (1) language, communication and misunderstandings; (2) navigating healthcare systems; (3) knowledge and understanding of NDDs and (4) cultural norms and beliefs. (1) *Language, communication and misunderstandings* reflected how communication barriers, including limited English proficiency, lower health literacy and culturally-shaped communication styles, made it difficult for families to clearly express concerns. HCPs noted these challenges frequently led to misunderstandings and delayed referral. (2) *Navigating healthcare systems* depicted the complexity of navigating NDD pathways. HCPs felt many families lacked knowledge of how services worked or how to advocate within them, which reduced access even when needs were recognised. (3) *Knowledge and understanding of NDDs* emphasised the central role of caregiver understanding of NDDs. HCPs recognised how limited awareness impeded caregivers’ ability to identify early signs, seek timely help and provide the information required for assessment. (4) *Cultural norms and beliefs* captured how cultural and religious beliefs, including stigma, norms around child behaviour and differing views on mental health and interacting with systems, shaped whether families sought, accepted or continued with support.

### Potential solutions

Throughout both survey and focus group findings, HCPs identified a range of strategies for engaging minority ethnic families seeking NDD support. Content analysis of the free text data and thematic analysis of focus groups data provided examples of these (see table 3).

**Table 3:** Strategies and suggestions used by healthcare professionals to help overcome identified barriers by ethnic minority families accessing NDD support services

Identified category	Strategies used	Description/examples
1. Language, communication and misunderstandings	The use of translator/interpreter services	I find using face-to-face interpreters/translators more valuable than telephone calls and video calls as they are able to pick up on the nuances in the conversation and non-verbal communication.
	Cultural competency training to understand differences	Cultural/religious beliefs are explored to unpick whether a ‘symptom’ is constitutional or the result of the child not fitting in with culturally accepted norm
2. The challenges of navigating healthcare systems	Providing clear information about services	I offer translation/interpretation, have reports translated and try and use simpler language to describe complex medical things with them to support understanding.
	Longer or multiple appointments	We offer longer appointments if needed or take more appointments to do assessment if needed to ensure everyone has time to fully understand.
3. The importance of having knowledge and understanding of NDDs	Community-based information	We engaged with the Black majority Christian churches and with the mosques; there are two or three mosques locally.
	Adapting language used to explain NDDs	I consciously change the way I describe symptoms. The use of language is important to build trust.
4. The influence of cultural norms and beliefs	Staff representation can help	Parents/clients welcome the opportunity to speak with a clinician of a similar background which triggers general queries around the young person’s interventions from the service.
	Connection and trust with local communities	I’m a more permanent member of the team and seen more regularly; families are often more trusting of my professional opinion if I raise issues/concerns relating to neuro-differences

NDD, neurodevelopmental disorders.

## Discussion

Using both qualitative survey and focus group data, and quantitative survey data from HCPs, this multiple-methods study found four interconnected barriers that limit minority ethnic families’ access to NDD services from HCP perspectives: (1) language and communication challenges; (2) difficulties navigating complex and fragmented healthcare systems; (3) limited caregiver knowledge and understanding of NDDs and (4) cultural norms, beliefs and stigma that influence help seeking. These findings can be understood through a socioecological lens, whereby barriers to access arise from the interaction of factors at multiple levels. At the individual level, limited caregiver knowledge of NDDs influenced recognition of need. At the interpersonal level, communication challenges and language differences affected interactions between families and professionals. At the community level, cultural norms and stigma shaped help-seeking behaviours. At the structural level, complex and fragmented service pathways limited access even where need was identified. These findings align with existing evidence that minoritised ethnic communities face additional, layered barriers to NDD services beyond those experienced by white families. Prior research has documented stigma, communication difficulties, mistrust and lack of cultural alignment as significant barriers to accessing child mental health services.^13 14^ This study extends that evidence by presenting, for the first time in England,^19^ how clinicians themselves perceive and interpret these barriers within NDD pathways. It is important to acknowledge that these themes reflect HCPs’ perspectives, which may be shaped by broader professional, cultural and systemic assumptions. As such, some interpretations risk being read as deficit-oriented if not carefully contextualised. A deficit orientation is problematic as this often frames those experiencing inequality as being at fault. Many of the identified ‘barriers’ can also be understood as arising from a misalignment between healthcare systems and the diverse ways in which families understand, communicate and respond to child development. For example, expectations around help-seeking, communication style or advocacy reflect system norms that may advantage families familiar with Western healthcare models.

A strength of this study is its multiple methods design, combining data from a large and diverse national survey with in-depth focus groups involving professionals across primary care, CAMHS, paediatrics and allied health. The sample reflected variation in ethnicity, clinical role, region and years of experience and PPIE involvement enhanced cultural relevance. It is important to note that the findings reflect HCPs’ perspectives on barriers for minoritised ethnic families, rather than those directly from families. While this was the intentional focus of the study; it may introduce bias. Survey responses were disproportionately from the East Midlands area of England (which is ethnically diverse), and the focus groups, while professionally diverse, were modest in size. The themes identified were broad, crosscutting concepts rather than likely artefacts of regional variation; we would anticipate is of interest to other westernised countries where the global majority is minoritised to improve understanding of what strategies have the most positive impact.

This study provides valuable insights into professional perspectives, while highlighting the need for research directly capturing minoritised ethnic families’ lived experiences across different regions and service models. Further work is needed to examine how structural redesign, such as simplified referral pathways, use of cultural advocates, improved interpreter services or coproduced community outreach might reduce inequities in practice. Quantitative evaluation of the impact of cultural competence initiatives, workforce diversification and targeted NDD education for caregivers is also warranted. These strategies also reflect a required shift in the focus from addressing perceived deficits in families to adapting services, so they better align with the strengths, values and contexts of the communities they serve.

## Clinical implications

Clinically, services should simplify referral pathways, minimise reliance on patient-led navigation and improve directional support to families and provide proactive support for history-taking and care coordination. Communication must be adapted to be culturally sensitive and accessible, with routine use of tailored explanations and support tools. Mandatory, ongoing, cultural competency training is needed to improve clinician responsiveness, while integrated cross-sector working can reduce fragmentation. Embedding community engagement in service design and commissioning is essential to ensure services are accessible, acceptable and aligned with population needs.

## Conclusion

This study underscores the need for system-level initiatives to ensure equitable access to neurodevelopmental services for ethnic minority CYP. Clinicians described how culturally shaped communication, limited health literacy, service complexity and stigma intersect to produce avoidable delays in identification and support. These insights highlight actionable priorities: streamlined referral systems, culturally informed practice and partnership with trusted community networks. Embedding these changes within national policy and workforce development strategies is critical to achieve equitable service access.

## Supplementary material

10.1136/bmjment-2026-302716 online supplemental file 1

10.1136/bmjment-2026-302716 online supplemental file 2

10.1136/bmjment-2026-302716 online supplemental file 3

10.1136/bmjment-2026-302716 online supplemental file 4

## Data Availability

Data are available upon reasonable request.

## References

[1] De Domenico C, Alito A, Leonardi G (2025). Children and Adolescents with Co-Occurring Attention-Deficit/Hyperactivity Disorder and Autism Spectrum Disorder: A Systematic Review of Multimodal Interventions. J Clin Med.

[2] American Psychiatric Association (2013). Diagnostic and statistical manual of mental disorders (DSM-5®).

[3] Roman-Urrestarazu A, Tyson A, Gatica-Bahamonde G (2025). Bayesian prevalence of autism and unmet special education needs in Chile in a sample of three million school-age children. Autism.

[4] Arnold LE, Hodgkins P, Kahle J (2020). Long-Term Outcomes of ADHD: Academic Achievement and Performance. J Atten Disord.

[5] Sigafoos J, Waddington H, Sigafoos J (2022). Early assessment and intervention: introduction to the special issue. Adv Neurodev Disord.

[6] Gallin Z, Kolevzon AM, Reichenberg A (2025). Racial Differences in the Prevalence of Autism Spectrum Disorder: A Systematic Review. J Autism Dev Disord.

[7] Chung W, Jiang S-F, Paksarian D (2019). Trends in the Prevalence and Incidence of Attention-Deficit/Hyperactivity Disorder Among Adults and Children of Different Racial and Ethnic Groups. JAMA Netw Open.

[8] Allen K, Trethewey SP, Mathews F (2024). Experiences of commissioning services for child and adolescent mental health in England (UK): a qualitative framework analysis. BMJ Open.

[9] McKenna K, Wanni Arachchige Dona S, Gold L (2024). Barriers and Enablers of Service Access and Utilization for Children and Adolescents With Attention Deficit Hyperactivity Disorder: A Systematic Review. J Atten Disord.

[10] Babalola T, Sanguedolce G, Dipper L (2025). Barriers and facilitators of healthcare access for autistic children in the UK: a systematic review. Rev J Autism Dev Disord.

[11] GOV.UK (2022). Population of England and Wales - GOV.UK ethnicity facts and figures. https://www.ethnicity-facts-%20figures.service.gov.uk/uk-population-by-ethnicity/national-and-regional-populations/population-of%20england-and-wales/latest/.

[12] Campbell-Stephens RM (2021). Educational leadership and the global majority.

[13] Place V, Nabb B, Gubi E (2021). Perceived barriers to care for migrant children and young people with mental health problems and/or neurodevelopmental differences in high-income countries: a meta-ethnography. BMJ Open.

[14] Stepanova E, Croke S, Yu G (2025). “I am not a priority”: ethnic minority experiences of navigating mental health support and the need for culturally sensitive services during and beyond the pandemic. BMJ Ment Health.

[15] Kapadia D, Zhang J, Salway S (2022). Ethnic inequalities in healthcare a rapid evidence review. https://www.nhsrho.org/wp-content/uploads/2023/05/RHO-Rapid-Review-Final-Report_Summary_v.4.pdf.

[16] Tabish SA (2024). Health care management: principles and practice.

[17] Kisa A, Kisa S (2025). Structural racism as a fundamental cause of health inequities: a scoping review. Int J Equity Health.

[18] Sapiets SJ, Hastings RP, Totsika V (2024). Predictors of Access to Early Support in Families of Children with Suspected or Diagnosed Developmental Disabilities in the United Kingdom. J Autism Dev Disord.

[19] Edbrooke-Childs J, Newman R, Fleming I (2016). The association between ethnicity and care pathway for children with emotional problems in routinely collected child and adolescent mental health services data. Eur Child Adolesc Psychiatry.

[20] Vincent BP, Maryam Z, Ali N (2025). Barriers and facilitators to accessing services for neurodevelopmental disorders among the carers of individuals from Black, Asian and minority ethnic groups in the UK: a scoping review. BMJ Open.

[21] Heer K, Rose J, Larkin M (2016). The Challenges of Providing Culturally Competent Care Within a Disability Focused Team: A Phenomenological Exploration of Staff Experiences. J Transcult Nurs.

[22] Henderson DX, Baffour TD (2015). Applying a socio-ecological framework to thematic analysis using a statewide assessment of disproportionate minority contact in the United States. TQR.

[23] Anguera MT, Blanco-Villaseñor A, Losada JL (2018). Revisiting the difference between mixed methods and multimethods: is it all in the name?. Qual Quant.

[24] O’Cathain A, Murphy E, Nicholl J (2008). The quality of mixed methods studies in health services research. J Health Serv Res Policy.

[25] Harris PA, Taylor R, Thielke R (2009). Research electronic data capture (REDCap)--a metadata-driven methodology and workflow process for providing translational research informatics support. J Biomed Inform.

[26] Eysenbach G (2004). Improving the quality of Web surveys: the Checklist for Reporting Results of Internet E-Surveys (CHERRIES). J Med Internet Res.

[27] Green J, Thorogood N, Melendez-Torres G (2025). Qualitative methods for health research.

[28] Bansal N, Karlsen S, Sashidharan SP (2022). Understanding ethnic inequalities in mental healthcare in the UK: A meta-ethnography. PLoS Med.

[29] Hsieh H-F, Shannon SE (2005). Three approaches to qualitative content analysis. Qual Health Res.

[30] Braun V, Clarke V (2019). Reflecting on reflexive thematic analysis. Qual Res Sport Exerc Health.

[31] Byrne D (2022). A worked example of Braun and Clarke’s approach to reflexive thematic analysis. Qual Quant.

[32] Östlund U, Kidd L, Wengström Y (2011). Combining qualitative and quantitative research within mixed method research designs: a methodological review. Int J Nurs Stud.

[33] Olmos-Vega FM, Stalmeijer RE, Varpio L (2022). A practical guide to reflexivity in qualitative research: AMEE Guide No. 149. Med Teach.

[34] Staniszewska S, Brett J, Simera I (2017). GRIPP2 reporting checklists: tools to improve reporting of patient and public involvement in research. Res Involv Engagem.

[35] GOV.UK (2023). NHS workforce. https://www.ethnicity-facts-figures.service.gov.uk/workforce-and-business/workforce-diversity/nhs-workforce/latest/.

