# Supplementary material for: Understanding inequities in accessing neurodevelopmental pathways: a multiple methods study investigating healthcare professionals’ views on access for minoritised ethnic families
Source: BMJ Ment Health. 2026 Aug 11;29(1):e302716. doi: 10.1136/bmjment-2026-302716 (PMC13475559; doi:10.1136/bmjment-2026-302716)
Supplement: Supplementary file 1 [file bmjment-29-1-s002.pdf]

Supplemental file 2 – Focus group topic guide and free text questions

Core Focus Group topic guide

1) How do you approach the initial assessment and diagnosis process with ethnic minority children and their families? Are there specific cultural considerations you take into account?
2) Do you think ethnic minority groups are under-represented in your service?
3) Do you think ethnic minority groups experience more barriers to accessing child neurodevelopmental services than White British?
4) What do you think these challenges/barriers are and how might they differ to White British groups?
5) How do you address these challenges?
6) Thinking specifically about stigma - How do you approach the topic of stigma surrounding neurodevelopmental disorders
7) within ethnic minority communities, and what strategies do you use to address and reduce stigma
8) Have you ever faced situations where there was a disconnect between the family's cultural beliefs and 'Western' mental health practices? How did you navigate these situations?
9) Do you think the role of the parent/carer is as a "gatekeeper" to the child accessing services?

- Does this differ depending on the age of the child?

10) Do you think ethnic minority groups have as good understanding of neurodevelopmental disorders as White British?
11) Can you share examples of successful strategies you've used to build trust and rapport with ethnic minority families during the treatment process?
12) Do you think there's a difference in ethnic minority families' willingness to accept:
    a) Diagnosis
    b) Medication
    c) Therapy (behavioural/psychological)
13) Do you think other characteristics are important in determining engagement with services or understanding of NDD?
14) What role do you believe community and cultural organisations can play in supporting ethnic minority families with child neurodevelopmental disorder issues? Have you collaborated with such organisations in your work
15) Can you describe any cultural or community-specific interventions or resources you have incorporated into your treatment plans that have been effective for ethnic minority children and their families
16) Have you received any training in how to engage ethnic groups?
17) Do you feel confident supporting ethnic minority families with NDD

Thinking about the healthcare system:

18) Have you encountered instances of cultural insensitivity or bias within the healthcare system when dealing with ethnic minority families? How did you respond to these situations
19) Does your SERVICE make any adaptions to support ethnic minority patients?
20) Have you ever used translators?
21) What adaptions would you like YOU or YOUR SERVICE to be able to offer?

Free text questions

a) Have you ever faced situations where you felt there was a disconnect between the family's cultural beliefs and Western mental health practices?
b) Have you worked with translators or interpreters to support ethnic minority groups with child neurodevelopmental disorder issues?
c) Do YOU make any adjustments to your appointments if they are with an ethnic minority parent (with a concern about their child)?
d) Do YOU make any adjustments to your appointments if they are with an ethnic minority young person (without their parent)?
e) Do you think cultural or community-specific interventions can play a role in supporting families?
