# Supplementary material for: Understanding inequities in accessing neurodevelopmental pathways: a multiple methods study investigating healthcare professionals’ views on access for minoritised ethnic families
Source: BMJ Ment Health. 2026 Aug 11;29(1):e302716. doi: 10.1136/bmjment-2026-302716 (PMC13475559; doi:10.1136/bmjment-2026-302716)
Supplement: Supplementary file 2 [file bmjment-29-1-s004.pdf]

Supplemental file 4 – Free text summary table

Summative content analysis results from the five free text survey questions

Response category	Frequency of response n/454 (%)	Example response
Challenges relating to navigating cultural or religious differences and perspectives. Misconceptions regarding mental health and NDDs	102/454<br><br>22.4%	*"They [Ethnic Minority Families] often believe their child is lazy, spending time with the wrong crowd, not religious enough etc so are less likely to be happy with treatment options as they don't believe anything to be fundamentally wrong with their child's mental health"*
Recognising the need to adjust language and communications around NDDs and mental health.	91/454<br><br>20.0%	*"I adapt and modify my language when identifying and discussing symptoms/features of their child's presentation and what this may mean. I do find that I do this more with families from minority backgrounds. If they [Ethnic Minority Families] don't use an NDD label, I often don't use a label either if I fear this will cause offense, upset or disengagement"*
Community engagement and involvement includes both positives and benefits, being both helpful and unhelpful at different times.	85/454<br><br>18.7%	"[I *think that] culturally or community-specific interventions can play a crucial role in increasing awareness of neurodiverse conditions within ethnic minority communities. These [neurodevelopmental disorders] conditions are often poorly recognized or misunderstood in these groups. Ethnic minority patients are often more receptive to information provided by their own communities than through conventional health services. Community members also tend to communicate in a*

		way that is more relatable and easier for these groups to digest, as they have built stronger rapport and have a deeper understanding of their needs."
Recognition of the importance and impact relationships and continuity of care have with ethnic minority communities.	69/454<br><br>15.2%	*"Reaching out to these communities with consistency will reduce barriers, expand a sense of inclusivity and genuine care. This begins with getting recognised leaders/celebrities of similar backgrounds onboard first."*
The pitfalls of translation and interpretation services: the impact of modality and quality of service.	62/454<br><br>13.7%	*"The interpreter was interpreting with their own well-meaning summary rather than translating, this led to some misunderstanding. [It can be] very hard where there may not be exact translations"*
Restrictions within services can limit professionals' ability to cater to ethnic minority families' individual needs.	45/454<br><br>9.9%	*"Our service is struggling with a huge waiting list for NDD assessments at the moment, and parents/carers are being asked to seek assessment via 'Right to Choose'. This is a massive barrier for some of our families, who have a different understanding of ND and the labels around this, especially if they have a language barrier."*

Note: NDD = neurodevelopmental disorder
