# Supplementary material for: Understanding inequities in accessing neurodevelopmental pathways: a multiple methods study investigating healthcare professionals’ views on access for minoritised ethnic families
Source: BMJ Ment Health. 2026 Aug 11;29(1):e302716. doi: 10.1136/bmjment-2026-302716 (PMC13475559; doi:10.1136/bmjment-2026-302716)
Supplement: Supplementary file 3 [file bmjment-29-1-s001.docx]

# Supplemental File 1:

Supplemental file 1: GRAMMS Checklist

Good Reporting of A Mixed Methods Study (GRAMMS) checklist

| Guideline | Section & Page |
| --- | --- |
| Describe the justification for using a mixed methods approach to the research question | Methods, pg. 4-8  Discussion, pg. 18-19 |
| Describe the design in terms of the purpose, priority and sequence of methods | Methods, pg. 4-8 |
| Describe each method in terms of sampling, data collection and analysis | Methods, pg. 4-8 |
| Describe where integration has occurred, how it has occurred and who has participated in it | Methods, pg. 4-8.  Findings, pg. 8-17 |
| Describe any limitation of one method associated with the present of the other method | Discussion, pg. 18-19 |
| Describe any insights gained from mixing or integrating methods | Findings, pg. 16  Discussion, pg. 18-19 |

O'Cathain A, Murphy E, Nicholl J. The quality of mixed methods studies in health services research. J Health Serv Res Policy. 2008;13: 92-98.

Supplemental file 1: CHERRIES Checklist

Checklist for Reporting Results if Internet E-Surveys

| Item Category | Checklist Item | Complete |
| --- | --- | --- |
| Design | Describe survey design |  |
| Institutional Review Board (IRB) approval and informed consent form | IRB approval | n/a |
|  | Informed Consent |  |
|  | Data protection |  |
| Developing and re-testing | Development and testing |  |
| Recruitment process and description of the sample having access to the questionnaire | Described open vs closed survey |  |
|  | Contact mode |  |
|  | Advertising the survey |  |
| Survey administration | Web/email |  |
|  | Context |  |
|  | Mandatory/voluntary |  |
|  | Incentives |  |
|  | Time/date |  |
|  | Randomisation of items | n/a |
|  | Adaptive questioning |  |
|  | Number of items |  |
|  | Number of screens (pages) | n/a |
|  | Completeness check |  |
|  | Review step | n/a |
| Response rate | Unique site visitor | n/a |
|  | View rate | n/a |
|  | Participation rate |  |
|  | Completion rate |  |
| Preventing multiple entries from the same individual | Cookies used | n/a |
|  | IP check | n/a |
|  | Log file analysis |  |
|  | Registration | n/a |
| Analysis | Handling of incomplete questionnaires |  |
|  | Questionnaires submitted with an atypical timestamp | n/a |
|  | Statistical correction |  |

Supplemental file 1 : GRIPP2 Reporting Checklist – Short Form

Guidance for Reporting Involvement of Patients and the Public (GRIPP2) checklist

| Section and Topic | Item | Section where reported |
| --- | --- | --- |
| 1. Aim | Report the aim of PPIE (patient and public involvement & Engagement) in the study | Abstract and methods |
| 2.Methods | Provide a clear description of the methods used for PPIE in the study | Contribution to the study design and conduct via monthly meetings and offline review tasks. |
| 3.Study Results | Outcomes—Report the results of PPI in the study, including both positive and negative outcomes | Online survey testing for clarity before going live to participants. Feedback on final study promotional posters. Feedback on question wording and inclusion of specific items in survey and focus group questions. Feedback on the narrative of results |
| 4.Discussion and Conclusion | Outcomes—Comment on the extent to which PPI influenced the study overall. Describe positive and negative effects | PPI ensured branching logic was sound for the online survey. PPI ensured study promotional materials were appealing and had inclusive wording. PPI provided guidance on recruitment strategies. PPI provided feedback on results. No negative effects observed or recorded |
| 5.Reflections | Comment critically on the study, reflecting on the things that went well and those that did not, so others can learn | PPI involvement was vital to provide lived experience viewpoints on what survey information was important to capture, as well as the wording of questions. The PPI group had representation from all ethnic minority groups targeted in the study and were able to provide feedback on visual aspect of study materials. Timings for meetings needed to fit in with school holidays or school timings as most members were parents. Study team needed to be flexible around PPI members caring and professional responsibilities and this can be a challenge. utilising personal connections also helped with recruitment. Overall, the study was more successful due to representative PPI involvement. |

S. Staniszewska, J. Brett, I. Simera, K. Seers, C. Mockford, S. Goodlad, et al. Research Involvement and Engagement 2017 3:1 2017-08-02 Vol. 3 Issue 1

Supplemental file 1 – Participant flow diagram
