# Supplementary material for: Understanding inequities in accessing neurodevelopmental pathways: a multiple methods study investigating healthcare professionals’ views on access for minoritised ethnic families
Source: BMJ Ment Health. 2026 Aug 11;29(1):e302716. doi: 10.1136/bmjment-2026-302716 (PMC13475559; doi:10.1136/bmjment-2026-302716)
Supplement: Supplementary file 4 [file bmjment-29-1-s003.docx]

Supplemental File 3

Participant Demographics

*Table 1: Demographic and background characteristics of healthcare professional participants*

| Demographics/Background  Survey N= 264 | Survey  N (%) |
| --- | --- |
| **Gender** |  |
| Female | 195 (73.9) |
| Male | 65 (24.6) |
| Other/prefer not to say | 4 (1.6) |
| **Age in years** (Mean, Range) | 43, 24-64 |
| **Ethnicity** |  |
| White | 169 (64.0) |
| Asian: Indian | 35 (13.3) |
| Asian: Pakistani | 10 (3.8) |
| Asian: Other | 10 (3.8) |
| Black (African & Caribbean) | 19 (7.2) |
| Mixed | 16 (6.1) |
| Other | 5 (1.9) |
| **Job role** |  |
| General Practitioner | 77 (29.2) |
| Clinical Psychologist | 23 (8.7) |
| Therapist | 21 (8.0) |
| Psychiatrist | 13 (4.9) |
| Paediatrician | 13 (4.9) |
| Nurse/Advanced Nurse Practitioner | 12 (4.5) |
| Assistant Psychologist | 11 (4.2) |
| Clinical Nurse Specialist | 8 (3.0) |
| Service Manager | 7 (2.7) |
| Clinical Director | 3 (1.1) |
| Social Worker | 2 (0.8) |
| Mental health counsellor | 2 (0.8) |
| Other | 72 (27.3) |
| **Region** |  |
| East Midlands | 91 (34.5) |
| Northwest England | 43 (16.3) |
| Greater London | 30 (11.4) |
| Yorkshire & Humber | 27 (10.2) |
| Southeast England | 27 (10.2) |
| East of England | 19 (7.2) |
| West Midlands | 12 (4.5) |
| Southwest England | 7 (2.7) |
| South Central | 6 (2.3) |
| Northeast England | 2 (0.8) |
| **Service worked in** |  |
| NHS- Primary care | 114 (43.2) |
| CAMHS | 91 (24.5) |
| Paediatric Services (community) | 28 (10.6) |
| Other community settings | 12 (4.5) |
| Education Settings | 5 (1.9) |
| Paediatric services (acute) | 4 (1.5) |
| Other | 10 (3.8) |
| **Participant self-reported proportion of their current clinical population being ethnic minority children and young people** | |
| 0% | 5 (1.9) |
| 1-9% | 57 (21.6) |
| 10-39% | 95 (36.0) |
| 40-64% | 47 (17.8) |
| 65-89% | 23 (8.7) |
| 90-100% | 5 (1.9) |
| Other | 32 (12.1) |
| **Number of years working in healthcare** |  |
| 10+ | 127 (48.1) |
| 7-9 | 22 (8.3) |
| 4-6 | 42 (15.9) |
| 1-3 | 51 (19.3) |
| Less than 1 year | 22 (8.3) |
| **Number of years working in current role (years)** |  |
| 10+ | 77 (29.2) |
| 7-9 | 16 (6.1) |
| 4-6 | 38 (14.4) |
| 1-3 | 93 (35.2) |
| Less than 1 year | 40 (15.2) |

Note: NHS = National Health Service, CAMHS = Child and Adolescent Mental Health Services

Job roles/activities undertaken by healthcare professionals

| Responsible for the following services for children and young people* | HCPs confirming this n (%) |
| --- | --- |
| Assessing mental health | 189 (72.0) |
| Screening for mental health difficulties | 168 (63.6) |
| Treatment for mental health (psychological/therapy/behaviour) | 128 (48.5) |
| Diagnosing mental health difficulties | 111 (42.0) |
| Treatment for mental health (pharmacological) | 73 (27.7) |
| Managing mental health services and see patients | 33 (12.5) |
| Managing mental health services, not currently seeing patients | 21 (8.0) |

*Caption *Categories were not mutually exclusive, so multiple activities were undertaken by healthcare professional participants*

*Comparison of GP vs Other HCP on their perceived barriers to NDD services for minority ethnic caregivers seeking support for their CYP*

| **Perceived barrier** | **GP N=77 (29% )** | **All other HCP N=187 (71%)** |
| --- | --- | --- |
| Language | 71 (92.2) | 168 (89.8) |
| Stigma | 71 (92.2) | 156 (83.4) |
| Discrimination Services/Staff | 35 (45.5) | 78 (41.7) |
| Discrimination Family | 64 (83.1) | 137 (73.3) |
| Understanding Access Services | 71 (92.2) | 153 (81.8) |
| Understanding Identity Symptoms | 59 (76.6) | 133 (71.1) |
| Supernatural/spiritual | 25 (32.5) | 63 (33.7) |
| Religious Beliefs | 36 (46.8) | 110 (58.8) |
| Distrust Healthcare Services | 34 (44.2) | 91 (48.7) |
| Lack of Belief in Western Treatment Options | 26 (33.8) | 64 (34.2) |
| Logistical Reasons | 49 (63.6) | 115 (61.5) |

*Comparison of longer service HCP vs shorter service HCP on their perceived barriers to NDD services for minority ethnic caregivers seeking support for their CYP*

| **Perceived barrier** | **Total years in service 7-10+ N=149 (56.4%)** | **Total years in service less than 1 year - 6 N=115 (43.7%)** |
| --- | --- | --- |
| Language | 137 (91.9) | 102 (88.7) |
| Stigma | 133 (89.3) | 94 (81.7) |
| Discrimination Services/Staff | 69 (46.3) | 44 (38.3) |
| Discrimination Family | 124 (83.2) | 77 (67.0) |
| Understanding Access Services | 130 (87.2) | 94 (81.7) |
| Understanding Identity Symptoms | 112 (75.2) | 80 (69.6) |
| Supernatural/spiritual | 55 (36.9) | 33 (28.7) |
| Religious Beliefs | 81 (54.4) | 65 (56.5) |
| Distrust Healthcare Services | 72 (48.3) | 53 (46.1) |
| Lack of Belief in Western Treatment Options | 55 (36.9) | 35 (30.4) |
| Logistical Reasons | 94 (63.1) | 70 (60.9) |
